# Lessons for the Implementability and Sustainability of the SURG-Africa Model of Malawi in Colombia: Comment on "Improving Access to Surgery Through Surgical Team Mentoring – Policy Lessons From Group Model Building With Local Stakeholders in Malawi"

**DOI:** 10.34172/ijhpm.2022.6974

**Published:** 2022-06-21

**Authors:** Jaime Hernán Rodríguez Moreno, Jesús Velandia, Diana Igua

**Affiliations:** Family Medicine Department, Universidad Pedagógica y Tecnológica de Colombia (UPTC), Tunja, Colombia.

**Keywords:** Health Management, Healthcare Policies, Implementation, Quality in Healthcare, Latin America

## Abstract

The development of models that allow improving the quality to achieve person-centered care is a challenge for any health system, especially in low- and middle-income countries, due to the economic difficulties inherent to the countries and to the cost involved in its implementation, which should be assumed by the states, avoiding that the economic burden is assumed by the population, and approaching the goal of universal health coverage. The availability of human talent and efficiency in the use of basic and specialized human talent is a necessity to improve safe access to health services, in this sense, the model proposed by SURG-Africa and whose sustainability in Malawi was evaluated, is an important reference for the establishment and sustainability of these models with other specialties and in other countries. Through this article, the elements of education, care model and financing for the implementation of the strategy in family medicine in the Colombian health system are explored.

Through a mixed methods study, Broekhuizen and colleagues^1^ evaluate the sustainability of the surgical team mentoring model in Malawi and given the relevance of health models based on low- and middle-income countries, they make it relevant to evaluate the scalability and reproducibility of these strategies in other countries. Quality in the provision of health services, defined as safe, effective, and people-centered care, includes the fulfillment of some essential attributes (timely, equitable, comprehensive, and efficient care) that lead to reduce the negative impact of health services, improving the effectiveness of healthcare services for the population.^2^ Next, the factors of the investigation by Broekhuizen and collaborators will be analyzed, for the implementation in the Colombian context.

## The Transfer of the Care Model

 The functioning of health service providers implies not only that they function adequately within them, but that their articulation with other institutions of different complexity is adequate. In this sense, the articulation between hospitals of lesser and greater complexity offers benefits in health outcomes and performance, lower mortality, lower costs, and symbiosis between reference (less complex) and reference (more complex) institutions. In order to optimize the benefits, the barriers identified in the implementation of the model of the study of the mentoring of the surgical team for the construction of group models with interested parties in Malawi should be intervened, which according to the social determinants of health can be classified as individual, interpersonal and contextual.

Individual barriers include inadequate monitoring of risk factors for the disease, poor self-care, low therapeutic adherence, complication of the initial pathology, user dissatisfaction with the health service, and psychological and work difficulties associated with the disease.^3^ The study shows that these factors occur due to disarticulation among the reference centers and exalts the complications in the patient’s pathologies due to an untimely remission or due to subjecting the patient to a non-required surgical intervention.

Interpersonal barriers include institutional deficit in supplies, infrastructure, regulations, and training for human talent in health; Furthermore, poor institutional coordination leads to unnecessary repetition of diagnostic studies, polypharmacy, confusion with the therapeutic plan, inadequate follow-up, deficient comprehensive care, and inappropriate care; caregiver overload or lack of family support in the process^3^; limiting the training of human talent in health for low complexity hospitals and the opportunity to access surgical processes.

Contextual barriers include non-compliance with public healthcare policies, economic unsustainability of the system, especially in southern countries, which have high poverty rates and a Gini coefficient with a tendency of 1 (perfect inequality); social dissatisfaction with services, poor culture of self-care, greater burden of disease and disability.^3^ In this study, the disarticulation between the program and national public policy limits its sustainability, since the state cannot finance it indefinitely, therefore, a challenge of the study is to find a solution to expand the program, demonstrate its profitability and self-sustainability over time.

 The determinants analyzed in the study in Malawi, the main barrier detected is the disarticulation between the implementation program of the study model in Malawi and the national health system. A similar situation occurs in other countries, especially in Latin America and Colombia, where the structuring of service networks working in an articulated and complementary manner from central hospitals to district or local hospitals, as proposed in the model evaluated, is a significant step forward in improving health outcomes for the population.

The articulation of models based on telehealth, with low cost and high diffusion tools such as WhatsApp, allow to strengthen the interaction between the members of the health teams, to support the resolution of complex clinical situations, improving patient health, improving the efficiency in the use of resources, and strengthening the confidence of the population in the health systems.^1^

Integrating these tools in the Territorial Model of Care in Colombia (MAITE), would significantly contribute to reduce barriers to access to specialized services, especially for those territories with difficult geographical access or where more immediate support is required to solve complex situations in local hospitals in the country and the displacement of the patient to another would delay important actions to improve their health condition, these interventions would make the implementation of this model, more timely and with more benefits for the population.^4^

## Mentoring and Training to Strength Family Physician’s Skills

Another perspective expressed by Broekhuizen et al^1^ is related to the mentoring that is done from hospitals of greater complexity to those of less complexity, in this sense, strengthening the capacities of health professionals in all areas of care is necessary and it improves the possibilities of reaching articulated institutions that improve the quality of care and especially patient safety.

 Based on the above, this mentoring model has been used in Colombia in different types of public and private institutions, and with different levels of accompaniment, there are experiences of synchronous support in the management of patients through telemedicine in intensive care, accompaniment in decision-making with the pregnant woman and more recently in a model that sought to support the training of specialists in family medicine for rural areas or areas with low population density.

In this last experience in the department of Guainía in Colombia, the Ministry of Health supported the training of specialists, where they in a high proportion of practice time was developed through mentoring by specialized professionals from the university that developed the program, facilitating their learning, but at the same time solving the main health problems of the communities.^5^

 However, a mentoring model between members of articulated hospitals has not been developed in Colombia, and the development of these mentoring models, which would allow the definition of complementary clinical management guidelines between local and central hospitals, would have a great impact on the health system in Colombia, improving the relationship between health professionals, trust between care teams and trust between the population and hospital professionals.

 The articulation of this model would be based on regional characteristics, as proposed in the Malawi model, where the central hospital in the region would support the smaller hospitals or care centers and provide them with clinical mentoring to ensure complementarity between the large hospital and the smaller and less-resourced one, improving the hospital’s capacity, the network and the results for the population.

Accompanying the care processes from the institutions of greater complexity will certainly achieve that all the members of the health systems obtain benefits, it will lower paying costs by making a more efficient use of resources, it will improve health results for the population, allowing more timely care, with a higher level of resolution of needs, improving the safety of care, reducing the number of complications and will result in a better patient experience within the care process.^6^

## Financial Sustainability of the Model

 The introduction of care models based on synchronous or asynchronous tools that favor access to health services, reverse the burden of spending from the community to the health service provider, in current models of care, the patient is the one who must approach institutions to obtain services, while in telehealth models, tools for patient use must be provided by the institution that delivers the services. These models result in higher health spending for the systems, which include not only the generation of tools, but also the maintenance and operation of services in the short and medium term.

As indicated by Broekhuizen and collaborators,^1^ the sustainability of these models must not only be ensured through fixed budgets, but also be guided by results-based payment mechanisms, which allow generating positive incentives with clinical results. Previously defined among service providers and health system funders, these results can be associated with general indicators (readmission rates) or disease-specific indicators such as optimal laboratory parameters (glycosylated hemoglobin in diabetic patients).^7^

The definition of the results to be evaluated for the definition of the payment mechanism must consider those that are directly related to the health service provider and that do not depend on externalities such as those attributable to the patient, geographical factors, or factors. Cultural or external barriers to the health system, in this way the definition of these payment mechanisms will bring more favorable performance and will lead to a sustainability of the strategy in the long term.^8^

 The financial sustainability of this type of program requires a necessary evaluation of the innovations and the impact on patient outcomes, thus showing the impact in economic terms through cost-effectiveness studies that show the outcomes. From the perspective of the system, the payer and the patient, and that they become the social agreement with these communities from the health system.

## Conclusion

 The evaluation of success factors, barriers and facilitators for the implementation of healthcare strategies, which aim for the population to obtain better health results, a better experience in care and for the system to use more Efficient resources is a need for all countries and especially those of low- and middle-income. Implementing a model articulated not only from the policies and regulations in Colombia, but also through the implementation at the level of health teams, with educational tools, based on clinical care and with support through widely disseminated mechanisms such as WhatsApp or other instant messaging systems. The SURG-Africa model, is a reference point to ensure that the policies adopted at the country level are put into practice by the institutions that are part of the system, with simple and highly efficient tools.

## Ethical issues

 Not applicable.

## Competing interests

 Authors declares that they have no competing interests.

## Authors’ contributions

 JHRM: Analysis and interpretation of data, drafting of the manuscript, supervision. JV: Drafting of the manuscript, analysis and interpretation of data, critical revision of the manuscript for important intellectual content. DI: Drafting of the manuscript, analysis and interpretation of data, critical revision of the manuscript for important intellectual content.

## Disclaimer

 The comments in this manuscript reflect only the personal position of the authors and do not represent the views of the university.
